# Success factors when implementing a structured support model for systematic work environment management in operating departments: A case study from Sweden

**DOI:** 10.1111/jonm.13812

**Published:** 2022-10-05

**Authors:** Erebouni Arakelian, Fredrik Molin, Magnus Svartengren

**Affiliations:** ^1^ Department of Surgical Sciences Uppsala University Uppsala Sweden; ^2^ IPF The Institute for Organizational and Leadership Development at Uppsala University Uppsala Sweden; ^3^ Department of Medical Sciences, Occupational and Environmental Medicine Uppsala University Uppsala Sweden

**Keywords:** implementation, nurse, perioperative, structured work model, work environment

## Abstract

**Aim:**

The study aimed to investigate how departments in a Swedish hospital worked with a structured support model between the sessions and what they identified as success factors.

**Background:**

To improve the work environment in a Swedish hospital, a structured support model for systematic work environment management was implemented in operating departments. The structured work starts with sending a web‐based, open‐ended, anonymous questionnaire to all employees. In response, employees describe how they perceive their work environment ‘right now’. Next, a session is held where employees' viewpoints are discussed, and areas of improvement are agreed upon. Action plans are created between the sessions, and the employees start working with their plans with support from their managers. Implementing new models takes time and requires efforts from employees and managers.

**Method:**

A case study was conducted, including three operating departments within a perioperative organization in a university hospital in Sweden. The participating departments had used the model without interruption during the Covid‐19 pandemic 2 years after implementation, and they had created a customized working method. Three first‐line managers were interviewed, and 22 action plans, 21 workplace meeting notes and two presentations were analysed using thematic analysis.

**Results:**

The results are sorted under three main thematic headings: Experience of results and benefits, Marketing and cheering on and Making adjustments and making the model one's own. The results from the action plans and workplace meetings indicated that the employees had discussed problems with cooperation, work organization and how to treat each other.

**Conclusion:**

Human factors, such as support, encouragement, seeing the benefits, allowing for time and respecting each other can facilitate and contribute to the implementation and success of a new model.

**Implications for Nursing Management:**

The main finding of the study indicates that with a structured way of working, and with the participation of the employees in the systematic work environment work, the employees contributed with constructive suggestions for improvement. This, in turn, contributed to reducing the workload for first‐line managers. In addition, when working with a structured model, deficiencies in the workplace were identified, which triggered an improvement process in the participating hospital departments.

## 1 BACKGROUND

According to Swedish legislation, in workplaces, employees and their managers are obligated to create and maintain a healthy work environment (Swedish Work Environment Authority, 2001, 2015a, 2015b). Despite regulations, there are still organizations in Sweden that do not manage their systematic work environment work properly (Frick, 2014). The demand on health care is high and increasing in the world, especially in Sweden, and it is a challenge to recruit and keep personnel in the healthcare sector. Systematic work environmental management should improve the development and increase the attraction for employees to work and remain within the healthcare sector. A structured way of working with work environment issues should include employees and stimulate them to find constructive solutions to what they identify as environmental challenges. In turn, managers can be relieved from findings all answers.

### 1.1 Work environment in a perioperative context

Perioperative context (pre‐, before; intra‐, during; and postoperative, after surgery) means working in a high‐tech work environment, and employees can be affected by poor working conditions and poor work environment (Logde et al., 2018), which, in turn, affects the quality of patient care (Aiken et al., 2012; Woo et al., 2017), and patient safety (Logde et al., 2018). Wålinder et al. (2018) describe that 30% of almost 1000 perioperative employees (including nurse anaesthetists and operating room nurses) had sometimes thought of leaving their position (during at least 1 month in the last year). A shortage of nurses is a general problem (Drennan & Ross, 2019), especially in perioperative contexts where it may lead to surgeries being cancelled. Logde et al. (2018) described that nurse anaesthetists and operating room nurses left their jobs for several reasons: the nurse managers' betrayal and dismissive attitude, inhumane working conditions and colleagues' dismissive behaviour. In contrast, factors that contributed to nurses staying in perioperative contexts were organizational stability with low staff turnover, good spirits between colleagues, to recognize everyone's equal value at the workplace, sustained development in one's own profession and a humane nurse manager who helped employees to develop (Arakelian et al., 2019).

### 1.2 Using a structured support model in a perioperative context

**FIGURE 1:**
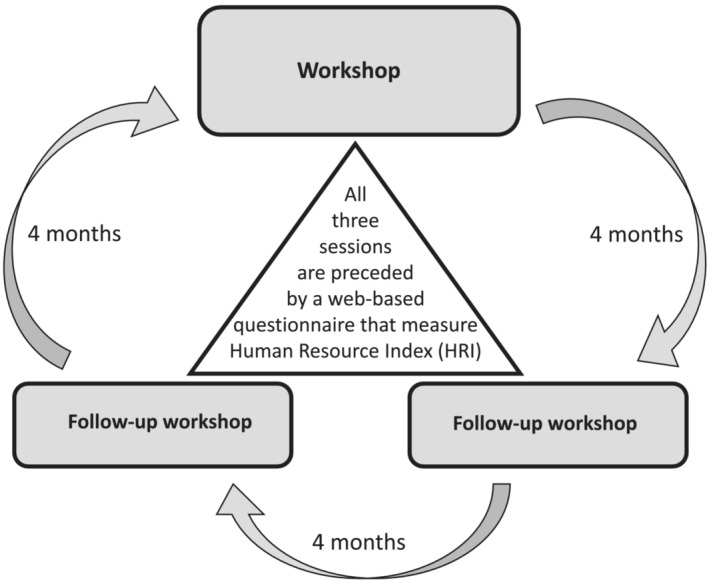
The yearly cycle of the Stamina model

A structured support model was described by Svartengren and Hellman (2018) for systematic work environment management. The model is flexible and can be used with a successful outcome, both in municipalities and in a perioperative setting in hospitals with their unique working context. Working with the model, employees and their leaders are engaged in managing their work environment in a structured and systematic manner. There are three to four cycles in the model annually. A cycle starts with sending a web‐based, open‐ended, anonymous questionnaire to all employees. In response, employees describe how they perceive their work environment ‘right now’. Next, a session is held where employees' viewpoints are discussed, and areas of improvement are agreed upon. Action plans are created between the sessions, and the employees start working with their plans with support from their managers. Each cycle is to be repeated three to four times annually. There is a built‐in process feedback measurement in the model called Human Resources Index (HRI), which can be measured at any given timepoint as a single measure (see Figure 1). Changes in HRI value can be used to evaluate how the work environment changes over time (Molin et al., 2021). A longitudinal quantitative study by Arakelian et al. (2021) reported a positive trend in HRI, concluding that a structured support model is a helpful tool, and HRI is a simple measure to follow‐up on work environment processes.

### 1.3 Implementing a new model in new settings and the role of leadership

Hojberg et al. (2018) identified four dimensions for a successful implementation of an intervention: a supportive organizational climate, a workplace with mutual goals for employees, and an ‘attractive’ intervention, which can be adapted to the workplace. In contrast, Martinsson et al. (2016) indicate that long‐term implementation of interventions in organizations tend to fail if they do not produce rapid results. Moreover, finding a suitable intervention for a specific workplace is a challenge that needs to be addressed, as interventions work differently in various contexts (Goodridge et al., 2015; Greenhalgh et al., 2015). It is important to consider that interventions that are found to be effective in research may not be successful in practice (Hojberg et al., 2018). However, there is still a knowledge‐gap regarding why an intervention is successfully implemented in several workplaces in an organization but fails to succeed in other workplaces in the same organization.

Leaders have an important role in the implementation process. Mann (2009) argued that 80% of the effort in implementation depends on changing the leaders' mindset, their practices and behaviours, as they set the tone for their employees. Mackenzie and Hall (2014) emphasize the leaders' important role in creating a vision and facilitating the understanding of the benefits of the intervention for the individuals and the organization as a whole. Leaders may have different roles during different phases of implementation. They may support implementation by motivating others, establishing goals and removing barriers. Some leaders work by delegating duties, or by ‘modeling the way’. Arakelian et al. (2020) pointed out that the ‘culture’ of the organization, and the definition of the role for managers and the employees were important when implementing a structured support model in a perioperative setting. The results emphasized that there had to be a ‘paradigm shift’ in the role of employees, who more likely have a passive role, while managers are the active part in the implementation process. The managers were described as role models, the ones who the employees followed. Also, they needed to take a step back and allow the employees to step forward and take greater responsibility in the implementation process. Role description, goal definition, timely feedback and sticking to one model were defined success factors. Molin et al. (2020) who studied first‐line managers' experiences of implementing a structured support model in Swedish municipalities reported similar results. They found that despite managers' experiences of discomfort when giving the responsibility of working with work environmental issues to employees, they were impressed by their employees' success. Managers balanced between being quiescent and, at the same time, actively monitoring progress in the work.

Thus, the work environment in a perioperative setting places specific demands on the employees. The structured support model was implemented in perioperative settings in a university hospital in Sweden as a continuation of a larger project (Svartengren & Hellman, 2018) to support work environment management. This study investigates success factors when implementing and using the model.

## 2 AIM

The study aimed to investigate how departments in a Swedish hospital worked with a structured support model between the sessions and what they identified as success factors.

### 2.1 Study questions

1. Which problems are identified in the action plans?
2. How do the groups work with their problems between the sessions?
3. What are the success factors in working with the process, according to the first‐line managers?

## 3 METHODS

This study was performed as a case study (Yin, 2009) with a qualitative and prospective design, as part of a larger research programme performed in municipalities in Sweden on work environment (Svartengren & Hellman, 2018). It is a continuation of the original protocol in a new context, in a perioperative setting within a hospital.

### 3.1 The case

The included hospital was a university hospital in central Sweden, with approximately 8000 employees. The perioperative department consisted of operating departments, an intensive care department, postoperative departments and a sterile processing department and had approximately 900 employees, of which approximately 500 were introduced to the structured support model. The inclusion criteria were departments that continued working with a structured support model through and after the Covid‐19 pandemic. Departments that interrupted their work with the model were excluded. The case includes three operating departments within a perioperative organization in a university hospital in Sweden. The common denominator was that 2 years after implementation, all three departments out of nine had used the model without interruption during the Covid‐19 pandemic, and that after a successful implementation of the model, they had created a customized working method. The remaining six departments interrupted their work with a structured support model due to the Covid‐19 pandemic. Thus, participating departments were sampled using a purposive sampling technique (Giacomini & Cook, 2000). The three departments had also shown a progression in the HRI measures (See Table 1). Table 2 provides information on the participating departments. Department B has different management responsibilities for operation room staff and anaesthesia staff, meaning that operating room staff did not participate in structured support model sessions. In other words, in Department B, only anaesthesia staff completed the sessions/cycles.

**TABLE 1:** Human Resources Index measure for the participating departments

	t1	t2	t3	t4	t5
**Department A**	47	58	68	N/A	N/A
**Department B** ^a^	37	60	60	70	N/A
**Department C**	49	55	63	66	78

*Note*: t1: baseline measure; t2: approximately 6 months after implementation; t3: approximately 12 months after implementation; t4: approximately 18 months after implementation; and t5: approximately 24 months after implementation.

^a^ Change of management between time‐point one and time‐point two.

**TABLE 2:** Participating departments in the study

	Department A	Department B^a^	Department C
**Number of employees**	45	30	Approx. 30
**Staff functions**	Nurse anaesthetists Operation room nurses Assistant nurses Anaesthesiologists	Nurse anaesthetists Assistant nurses Anaesthesiologists	Nurse anaesthetists Operation room nurses Assistant nurses Anaesthesiologists
**Manager experience**	11 years	3 years^b^	3½ years

^a^ The department has different management responsibilities for operation room staff and anaesthetist staff.

^b^ Change of management between session one and session two (i.e., baseline and 6 months after implementation of the structured support model).

### 3.2 Data collection and the participants

The departments' first‐line managers were invited to participate in the study, and all three accepted. Department B had a change of management between session one and session two (i.e., baseline and 6 months after implementation of the structured support model). The first line manager in Department B who worked with the structured support model after baseline was invited to participate, which she accepted. After obtaining informed consent, an interview was conducted face‐to‐face with one of the participants and through live video call with two of the first‐line managers between October 2021 and January 2022. The interview lasted between 25 and 60 (mean 45) min. All three interviewees were women, between 39 and 52 (mean 46) years of age and had three to 11 (mean 5.8) years of experience as nurse managers. In addition to interviews, 22 action plans (from Departments A and B), 21 workplace meeting notes (from Department C) and two PowerPoint presentations (from Departments A and C) were collected and analysed. Department A was provided with three action plans, one PowerPoint presentation and one interview. From Department B, we had 19 action plans and one interview, and from Department C there were 21 workplace meeting notes, one PowerPoint presentation and one interview.

### 3.3 The interview guide

The interview guide for this study included two main areas, that is, the process of implementing the structured support model (working with the action plans between the meetings) and the success factors for the implementation process, according to the managers (Table 3). Probing questions were used to get in‐depth information.

**TABLE 3:** The interview guide

Initial questions	How did you start working with Stamina model‐ at the starting point? What were your thoughts and your employees' thoughts about how to work with the Stamina model and your action plans? Please tell me about your employee group and their reactions about the model.
Main questions	When you started to work with the Stamina model, how did you work with your action plans between the meetings? (Knowing the fact that subgroups had started to work actively with action plans) how did you come up with the idea of setting up a subgroup of employees to work with your action plans between the meetings? What would you say were the challenges to work with the action plans between the meetings? How did you work with the action plans between the meetings? How did you influence the model to adapt it to your needs? What was the next step in working with the action plans in your opinion?
Probing question	Please tell me more.
	Can you give an example?
	What do you mean?

### 3.4 Data analysis

Thematic analysis of the interview text was performed in accordance to (Braun & Clarke, 2006) in several steps. The interviews and the text from the action plans were analysed separately. First, the texts were read through to grasp the whole meaning. Second, a first coding was performed separately by authors EA and FM. Subsequently, the two authors discussed the coding. Third, the provisional codes and the text sections that were linked to the codes were reviewed again. The codes were refined based on consistency and agreement between the code and the text sections. Sub‐codes that were irrelevant to the aim of this study were removed. Thereafter, themes were created, based on similarities and differences in the codes. Finally, the content in the themes was described, which are presented with quotes. The text from the action plans, workplace meeting notes and presentations, with focus on the manifest content of the text and study question 1, was analysed with content analysis according to Elo and Kyngäs (2008).

To ensure trustworthiness and credibility of the findings, the analysis process went back and forth between coding and the interview transcripts in several steps, as described previously (Nowell et al., 2017; Shenton, 2004). The analysis was discussed within the research group on several occasions. This case study has a limited scope because of its specific context. In our study some parts of the results/themes repeated themselves and some new information was discovered.

### 3.5 Ethical considerations

The study followed the Declaration of Helsinki regulations (World Medical Association, 2013) and local ethical guidelines and regulations (Centrum for Research Ethics and Bioethics, 2018). It was approved by the Swedish Ethical Review Authority (Dnr 2019‐00948).

## 4 RESULTS

### 4.1 First‐line managers' perspectives on implementing and working with the structured support‐model

The results are sorted under three main thematic headings: Experience of results and benefits, Marketing and cheering on and Making adjustments and making the model one's own.

#### 4.1.1 Experience of results and benefits

The managers appreciated the results from the questionnaire part of the model, which were deemed more useful than traditional employee surveys because they were closer to the daily operations of the department. ‘I think it was fun to work with [the model], and it was closer to our reality than an employee survey’ (Manager 2).

All three managers stressed the importance of continuity when working with the model and sticking to the suggested way of working to make improvements in the long run. ‘The employees feel more involved and come up with suggestions. You work together in the work group, and I think that this is important to make it work in the long run’ (Manager 2).

The managers also stressed the importance of the employees seeing the results and benefits of using the model. One manager stated that a result from using the model was the creation of specific occupational groups. This was a concrete development from the discussion initiated by the results from the questionnaire. Having a short time‐period between the questionnaire and the processing of the results was also emphasized. Seeing concrete results and improvements from the model was viewed as important in motivating the staff to continue using the model and to avoid a negative climate between different staff functions. One manager made it clear to the staff that those positive outcomes were a result of working with the model: ‘When the staff see a concrete change, I always refer to the model’ (Manager 1).

#### 4.1.2 Marketing and cheering on

The managers describe the importance of cheering on and encouraging the groups, which were viewed as success factors. They supported the work groups by being available and creating the right conditions for the work groups by making time in the schedule for meetings, which made the employees feel they were recognized and not ignored. ‘I was there as a support, and they never felt alone. I was there’ (Manager 3).

Another type of support was referred to as lobbying for the model. ‘What I did was to market the model as a work tool that was important and helpful and to help my employees to put words to their feelings’ (Manager 1). This manager expressed that there was a negative attitude from the staff at the beginning of the process, and therefore he tried to talk positively about the model. ‘It was not positive the first time/ … /Then I started to cheer them on during the work in the group’ (Manager 1). The manager started to support the group by giving positive comments and encouraging the employees to fill in the web questionnaire to get an even better basis for suggested improvements.

#### 4.1.3 Making adjustments and making the model one's own

The first‐line managers described adjusting the model, making it their own and not following the suggested work process exactly. This was identified as a success factor because it allowed for the groups to work with the model without major disruptions in the department's ordinary work schedule. It also made it easier for the manager to get the groups to accept the model. One manager stated: ‘According to the concept, this is something you should work with at every meeting, but I said [to the group] that this is the way *we are* working with the model. We decided to keep it simple’ (Manager 3).

Two managers had created separate work groups, with a rotating participation that prepared the results from the web‐based questionnaire and presented suggested actions to the group at a later stage. This adjustment allowed the groups to work more efficiently with the model. It seemed that news of this particular adjustment had spread between the departments when the managers of the groups had participated in common manager meetings. The suggested timeframes for the sessions were also adjusted depending on time available at the departments, by all three managers.

### 4.2 Action plans and workplace meetings notes and presentations

The results from the action plans and workplace meetings indicated that the employees had discussed problems with cooperation, work organization and how to treat each other.

### 4.3 Theme 1: Work organization and prerequisites for performing one's work tasks

#### 4.3.1 Having time for …

Having time for education or ‘to learn new things’, time for guidance in work situations, time for proper lunch breaks or not having enough time for lunch or coffee breaks were described in action plans as challenges that the employees wished to improve. Staff members had daily reflections where they asked about how the employees' day had been. This could, for example, be performed in between the patients or at the end of the workday.

*‘We lack time for education and time to learn things (to increase work related competence). Education time needs to be planned into the staffs' work schedule’* (Department A)

#### 4.3.2 Efficient meetings

Having morning meetings were identified as a source of stress, as the time for preparing for patient care became limited. Hence, employees wanted to have structured and efficient meetings. Structure referred to setting up an agenda prior to the meetings, to have a start and finish time, and to have someone present to lead the meetings. It was also suggested that the patient care should start 30 min after the morning meetings, so that preparation for patient care could be done in a non‐stressful manner. It was important that as many as possible, including anaesthesiologists, could attend the meetings, and that roles and work tasks could be discussed and agreed upon. Brief team‐meetings in the operating rooms in the morning were identified as important to plan the work, reduce wasted time and streamline the work.

*‘More structured meetings are required on Wednesdays. We need to prepare ourselves before the meetings. It will increase the feeling of participation’* (Department B)

#### 4.3.3 Planning resources and operating capacity

Need for better planning of resources in the operating department and in the postoperative ward versus operating programme was identified as an area of improvement. ‘Heavy programmes’ or sudden/late changes to operating programmes had to be reviewed, for example, in weekly plans, especially when staff members became sick or when additional operating rooms were used.

*‘Better planning of resources in the operating programme and in the operating department (resources contra production). Planning of breaks (for the staff) during both in the morning and in the afternoon’* (Department C)

Divergence in desired or ‘invented’ operating time and the ‘real time it takes to operate’, including preparation and completion time, had to be looked over. The management was asked to share their thoughts and plans and provide support in structuring the operating programme and operating hours. To review routines and work tasks in the interprofessional teamwork in order to be on the same page needed to be improved.

*‘Heavy programs which need to be reviewed when staff become ill or when staffing is low. Think of each other, ask for and offer help when you can or need it yourself’* (Department B)

#### 4.3.4 Education

Educating staff members about different operating procedures was another action plan that the staff members desired to work with. Daily educations, mini lectures, review of device usage, presenting the latest research in current and relevant topics were mentioned. Also, it was suggested to have a specific person with a specific responsibility for working with staff education and continued skill development.

*‘Deepen our knowledge, to update ourselves in research articles and new findings, continuous education’* (Department B)

#### 4.3.5 Engaging in work environment management

Moreover, it was requested that staff members be informed about renovations taking place in the work environment and the need to move from one place to another. The staff member wanted to participate and suggested creating a group of delegates on such occasions and informing colleagues continuously. Input from colleagues in working with work environment management was important. In one department that had built a group with specific responsibility for work environment issues, the group encouraged colleagues to be involved and come up with suggestions for problems.

*‘The vision is that we should think about the work environment every day. Make suggestions to a group (at the workplace) on these topics about how we can work further, come up with suggestions for improvements*, etc. *It is everyone's work environment’* (Department B)

*‘A meeting with anesthesia and the surgery group together should be planned to talk through the common work environment. Calmer work environment is experienced in the department’* (Department B)

### 4.4 Theme 2: Respecting each other and using a respectful tone towards each other

‘Uncollegiate’ manners, ‘envy’ or being ‘grumpy’ were identified among colleagues, especially in stressful situations, which the employees wanted to improve. It was suggested that colleagues should treat each other with respect and create a sense of community among each other. Everybody should step in, to counter ‘grumpiness’ with better communication, remembering that it can be ‘one's turn next time’. Treating others as one wants to be treated was encouraged, thus contributing to a good atmosphere. These actions were especially important when work was stressful. Furthermore, it was important to accept and embrace each other's differences, and to talk *to* each other, instead of talking *about* each other. The employees themselves asked whether they were being nice to each other, suggesting they should give each other both positive and negative feedback individually and not in front of the entire group. Stress because of communication problems was presented in some action plans, without further development.

*‘Show each other appreciation. Gratitude can also be shown from above (from the managers to the employees). It is important to take responsibility and show collegiality and to respect each other. Do debrief‐ and to allow everyone to talk about less pleasant things. Counter whine with better and clear communication’* (Department A)

*‘Are we kind to each other? There still appears that people talk about each other ‐ what is the purpose!?! Raise each other and talk to one another, not about each other’*. (Department B).

## 5 DISCUSSION

### 5.1 Main findings

The findings show that when working with the structured support model, the staff identified several areas for improvement. An example was the need for effective and structured meetings and the importance of having time for reflection after a workday. Managers stressed the importance of being able to modify the model to suit the needs of their employees and of supporting the employees between workshops.

### 5.2 Experience of results and benefits

The managers describe of the importance both for the groups and for the managers to see early benefits from the model. This finding confirms previous literature regarding implementation and change. For instance, Kotter (1995) describes the importance of celebrating early wins and see early concrete results of a change effort.

### 5.3 Marketing and cheering on

One success factor when implementing the structured support model, described by the managers, was marketing the model so that the employees would understand how they could benefit from using it. Hojberg et al. (2018) pointed out the impact of making the implementation ‘attractive’ for those who had to work with it. In this study, the managers tried to show connections between the changes within the operating department and the structured support model and supporting their employees when they needed. Mackenzie and Hall (2014) and Mann (2009) emphasized the role and importance of leaders, to change their employees' mindset and modelling the way.

### 5.4 Making adjustments

Furthermore, modifying the model to meet one's own department's needs was important to succeed with the model, according to the first‐line managers. Adjustments and the possibility to modify the model are described in the literature as important factors when implementing workplace interventions because models need to be adjusted to different contexts (Greenhalgh et al., 2015). Both Arakelian et al. (2020) and Molin et al. (2020) explained the need to back down as a manager and leave room for employees to take an active role in working with the model. This is confirmed in this study, where independent groups of employees were created in two of the three departments to work actively with the structured support model.

An important finding here was the importance of allowing for time and making room in the organization for the employees to work with their action plans, to keep the model ‘alive’ and continuing over a longer period (more than 2 years). This was confirmed by the action plans, which describe the implementation's success from the employees' point of view. Also, the managers expressed that they created groups of employees and gave them time to work with their action plans, which is an obvious example of managers and employees working towards the same goal, leading to success.

### 5.5 Action plans

The results from the action plans show the broader engagement of the employees in workplace issues, big and small, from opportunities to educate themselves further, organizational balance between resources and tasks, engaging oneself in work environment issues, and respecting each other and everyone's profession. Main findings from the analysis of the action plans were the importance of work organization and stable prerequisites for performing one's task, and the importance of a respectful social climate among the employees. These results would indicate the importance of social climate factors of concern in the investigated departments. Examples of social climate factors in the literature are involvement, co‐worker cohesion, supervisor support, autonomy, task orientation, work pressure, clarity, control, innovation and physical comfort (Moos, 1994). Geue (2018) show the positive relationship between social climate and positive practices, which includes respect and treating one another with integrity. Wheelan (2015) highlights the importance of a positive climate among team members and hoe this may influence group development and team performance.

As Arakelian et al. (2020) emphasized, and the managers in this study mentioned, the structured support model was closer to the employee's everyday life compared to other models, engaging them to work with different issues. Even though the managers were sceptical towards the new model in the beginning, they could appreciate and see the benefits after working with it for almost 2 years. The latter was also confirmed by Molin et al. (2020) who showed similar results in Swedish municipalities. Looking at the department's way of working with action plans and their progression in the HRI, one can see a greater positive development in department B, which also stuck to the model to a greater extent (i.e., they stuck to writing action plans, and not workplace meeting notes). However, one should consider the fact that department B changed its first‐line manager between timepoint one and two, which can also contribute to the development in HRI.

Martinsson et al. (2016) confirmed that to expect rapid changes from new implementations may not be long lasting. Thus, implementation may take time, as was the case here, and there may be a need for a change in the ‘culture’ of the organization by a ‘paradigm shift’, as Arakelian et al. (2020) pointed out. Consequently, both managers and their employees may have to work more intensively with the goal of the implementation and with role descriptions and responsibilities of both managers and their employees, which takes time.

### 5.6 Limitations

This study focuses solely on departments that have used the structured support model during a prolonged time. There is thus a potential bias that the studied departments have a positive bias since they have used and adopted the model with good results. This may have influenced the findings in a positive direction.

Another limitation of the study is that it only includes a managerial perspective regarding the issue of how the work groups engaged between the sessions. Getting an employee perspective on this issue would be valuable for further study.

## 6 CONCLUSION

Human factors, such as support, encouragement, seeing the benefits, allowing for time and respecting each other, can facilitate and contribute to the implementation and success of a new model. Managerial support and ability to tailor and modify the model to the needs of the organization are also important.

## 7 IMPLICATIONS FOR NURSING MANAGEMENT

The take home message of the study is that with a structured way of working, and with the participation of the employees in the systematic work environment work, the employees contributed with constructive suggestions for improvement. This, in turn, contributed to reducing the workload for first‐line managers. In addition, when working with a structured model, deficiencies in the workplace were identified, which triggered an improvement process in the participating hospital departments.

## CONFLICT OF INTEREST

Magnus Svartengren (M. S.) has been Chairman of the Board of Directors for the Human Resources Institute AB, the organization that has owned the legal rights to the Human Resources Index measurement tool since 13 November 2020. However, this had no influence on the research design, analysis and results. The funders had no role in the design of the study; in the collection, analyses or interpretation of data; in the writing of the manuscript, or in the decision to publish the results.

## ETHICS STATEMENT

The study followed the Declaration of Helsinki regulations (World Medical Association, 2013) and local ethical guidelines and regulations (Centrum for Research Ethics and Bioethics, 2018). It was approved by the Swedish Ethical Review Authority (Dnr 2019‐00948).

## Data Availability

The data that support the findings of this study are available on request from the corresponding author. The data are not publicly available due to privacy or ethical restrictions.
